# Activation of TRPC1 Channel by Metabotropic Glutamate Receptor mGluR5 Modulates Synaptic Plasticity and Spatial Working Memory

**DOI:** 10.3389/fncel.2018.00318

**Published:** 2018-09-14

**Authors:** Sophie Lepannetier, Roberta Gualdani, Sabrina Tempesta, Olivier Schakman, François Seghers, Anna Kreis, Xavier Yerna, Amina Slimi, Marie de Clippele, Nicolas Tajeddine, Thomas Voets, Robin S. Bon, David J. Beech, Fadel Tissir, Philippe Gailly

**Affiliations:** ^1^Cell Physiology, Institute of Neuroscience, Université catholique de Louvain, Brussels, Belgium; ^2^Laboratory of Ion Channel Research (VIB-KU Leuven Center for Brain & Disease Research), Department of Cellular and Molecular Medicine, Katholieke Universiteit Leuven, KU Leuven, Leuven, Belgium; ^3^School of Medicine, Faculty of Medicine and Health, University of Leeds, Leeds, United Kingdom; ^4^Developmental Neurobiology Group, Institute of NeuroScience, Université catholique de Louvain, Brussels, Belgium

**Keywords:** mGluR, TRP channels, synaptic plasticity, spatial memory, hippocampus

## Abstract

Group I metabotropic glutamate receptors, in particular mGluR5, have been implicated in various forms of synaptic plasticity that are believed to underlie declarative memory. We observed that mGluR5 specifically activated a channel containing TRPC1, an isoform of the canonical family of transient receptor potential (TRPC) channels highly expressed in CA1-3 regions of the hippocampus. TRPC1 is able to form tetrameric complexes with TRPC4 and/or TRPC5 isoforms. TRPC1/4/5 complexes have recently been involved in the efficiency of synaptic transmission in the hippocampus. We therefore used a mouse model devoid of TRPC1 expression to investigate the involvement of mGluR5-TRPC1 pathway in synaptic plasticity and memory formation. *Trpc1^-/-^* mice showed alterations in spatial working memory and fear conditioning. Activation of mGluR increased synaptic excitability in neurons from WT but not from *Trpc1^-/-^* mice. LTP triggered by a theta burst could not maintain over time in brain slices from *Trpc1^-/-^* mice. mGluR-induced LTD was also impaired in these mice. Finally, acute inhibition of TRPC1 by Pico145 on isolated neurons or on brain slices mimicked the genetic depletion of *Trpc1* and inhibited mGluR-induced entry of cations and subsequent effects on synaptic plasticity, excluding developmental or compensatory mechanisms in *Trpc1^-/-^* mice. In summary, our results indicate that TRPC1 plays a role in synaptic plasticity and spatial working memory processes.

## Introduction

The canonical family of transient receptor potential (TRPC) proteins owns seven members (TRPC1 to TRPC7) that form homo- and/or hetero-tetrameric Ca^2+^-permeable, nonselective cation membrane channels (Minke and Cook, 2002; Owsianik et al., 2006). Based on sequence homology, this family can be divided into three subfamilies: the TRPC1, -C4, and -C5 subfamily, the TRPC3, -C6, and -C7 subfamily and TRPC2, which is a pseudogene in humans (Montell et al., 2002). In contrast to other TRPC channels that are able to assemble in homo- and hetero-tetramers, creating a variety of different channels, the function of TRPC1 as an ion channel is a matter of debate (Dietrich et al., 2014). Indeed, it is not sure that it can form homotetramers and function on its own physiologically but may rather be an important linker and regulator protein in heteromeric TRPC channel tetramers.

TRPC channels have diverse functions in the brain (Selvaraj et al., 2010; Bollimuntha et al., 2011; Vennekens et al., 2012). They are involved in neurite outgrowth and axon guidance (Greka et al., 2003; Li et al., 2005; Wang and Poo, 2005), in neural progenitor cells proliferation and differentiation (Li et al., 2012; Du et al., 2017) and in neuronal apoptosis or survival (Tai et al., 2009; He et al., 2016). TRPC channels are also able to modulate neuronal excitability and could promote excitotoxicity (Phelan et al., 2012, 2013; Narayanan et al., 2014). Recently, Broker-Lai et al. (2017) demonstrated that the TRPC1/4/5 complex is involved presynaptically in the efficiency of synaptic transmission in the hippocampus.

Various activation mechanisms have been proposed for TRPC channels, the most frequently described being the activation by G-protein-coupled receptors or receptor tyrosine kinases via phospholipase C-induced formation of diacylglycerol (DAG) and inositol trisphosphate (IP_3_) (Gee et al., 2003; Trebak et al., 2007; Lemonnier et al., 2008; Tai et al., 2008). For the TRPC1 isoform on which this study concentrates, the gating mechanisms seem particularly diverse (Nesin and Tsiokas, 2014). In cerebellar Purkinje cells, TRPC1 was reported to be activated by metabotropic glutamate receptor 1 (mGluR1) (Kim et al., 2003). This was, however, refuted by Hartmann et al. (2008), who demonstrate that TRPC3 (and not TRPC1) was responsible for slow synaptic potentials and mGluR-mediated inward currents. In hippocampal neurons, however, El-Hassar et al. (2011) showed that stimulation of group I mGluR triggered IP_3_-dependent Ca^2+^ waves that contribute to an SK channel-mediated transient hyperpolarization followed by a sustained depolarization. On the basis of its sensitivity to blocking antibodies, this depolarization was attributed to the activation of TRPC1/4/5 channels (El-Hassar et al., 2011). TRPC1 is also involved in store-operated calcium entry in cooperation with Orai1 channel and activated by STIM1, a sensor of Ca^2+^ contents in the endoplasmic reticulum (Ambudkar et al., 2007; Cheng et al., 2008; Louis et al., 2008; Kim et al., 2009; Tajeddine and Gailly, 2012; Ong and Ambudkar, 2017). TRPC1 can be directly activated by phosphatidylinositol 3,4,5-trisphosphate (PIP3), by phosphorylation by PKC (Ahmmed et al., 2004; Saleh et al., 2009) or through the hydrolysis of phosphatidylinositol 4,5-bisphosphate (PIP_2_) and/or concomitant generation of DAG (Albert, 2011) or other lipids such as sphingosine-1-P (Lepannetier et al., 2016).

The present study is focused on the role of TRPC1 in the hippocampus and on its activation mechanism. We generated a genetically modified mouse model in which *Trpc1* gene was inactivated and noticed a specific impairment of spatial working memory and fear conditioning in *Trpc1^-/-^* mice. It is widely accepted that synaptic plasticity, the ability of synapses to strengthen or weaken over time in response to the activity pattern, reflects the processes that occur during formation and storage of memories. We therefore evaluated the involvement of TRPC1 in synaptic plasticity, in particular in long-term potentiation (LTP) and in long-term depression (LTD). To address possible brain developmental differences induced by the lack of expression of *Trpc1* gene, we also made use of Pico145 a selective inhibitor of TRPC1/4/5 channels recently described (Rubaiy et al., 2017a,b; Just et al., 2018).

## Materials and Methods

### Animals

All animals were housed and handled in accordance with European guidelines and approved by the animal ethics committee of the Université catholique de Louvain. This study was performed on male 2 to 4-month-old mice. At appropriate experimental time points, all animals were anesthetized and sacrificed.

### Generation of Trpc1 Knockout Mice

Embryonic stem cells containing the gene trap vector (*Trpc1*^*tm*1*a*(*EUCOMM*)*Hmgu*^) integrated into the exon 2 sequence of the *Trpc1* gene was obtained from International Mouse Phenotyping Consortium. The vector included a *LacZ* reporter gene, which, after integration, was under the control of the *Trpc1* promoter. Recombinant AK7 embryonic stem cells were injected into C57BL/6J blastocysts. The embryos were transferred into pseudopregnant CD1 mice. The chimeric males were bred with C57BL/6 females. Agouti mice harboring the selection and LacZ cassettes and (i.e., the KO first allele) were used to monitor the *Trpc1* expression. They were also mated with ROSA-*flp* females to have the selection cassette excised. The so obtained mice have the second exon of *Trpc1* flanked with *loxP* site. Breeding these mice with PGK-Cre recombinase mice line allowed to obtain a constitutive *Trpc1* knockout mouse line. This mouse line was tested for the presence of the disrupted *Trpc1* allele by PCR, using genomic tail DNA. Heterozygous mice were further bred to obtain homozygous mice on a mixed genetic C57BL6/129S1/Sv background. Heterozygous transgenic mice and their WT littermates were identified by PCR genotyping. In order to achieve temporally controlled somatic mutagenesis specifically in neurons of the forebrain region, the *Trpc1^lox/-^* mice were crossed with mice expressing the CreERT2 fusion protein under control of the regulatory elements of the CaMKIIα gene (CaMKCreERT2 transgene)(Erdmann et al., 2007; Schonig et al., 2012).

### Histology

Homozygous mice (*Trpc1^lacZ^*) were anesthetized and transcardially perfused with 4% paraformaldehyde in PBS. Brains were isolated, cryoprotected, and frozen sectioned (16 μm) in sagittal plane. For β-galactosidase activity detection, sections were fixed in 2% glutaraldehyde, washed, and stained 72 h at 37°C in PBS supplemented with 20 mM K_3_Fe(CN), 20 mM K_4_Fe(CN)_6_, 2 mM MgCl_2_, and 1 mg/mL X-gal. They were then washed and counterstained with Nuclear Fast Red. No staining was detected on sections from wild-type mice treated in the same conditions. A Leica SCN400 scanner acquired the pictures of complete sagittal brain sections.

### Western Blotting

Hippocampi were collected in RIPA buffer (25 mM Tris HCl pH 7.6, 150 mM NaCl, 1% NP-40, 1% sodium deoxycholate, and 0.1% SDS). The samples were clarified by centrifugation at 14000 g and the protein concentration was determined using bicinchoninic acid assay kit (BCA; Thermo Scientific). Samples were heated for 10 min at 70°C. 10 μg of protein for each sample was loaded on a 7% SDS-polyacrylamide gel, transferred to a nitrocellulose membrane. After blocking with 5% non-fat milk, membranes were probed overnight with anti mGluR5 (1/1000, Cell Signaling), anti-GluA1 (1/500, Merck), anti-GluA2 (1/1000, Merck), anti GluN2A (1/250, Merck), anti-GluN2B (1/500, BD Biosciences) or anti-β tubulin (1/10000, Neuromics). Membranes were then incubated with secondary antibodies coupled to peroxidase (Dako) and peroxidase was detected with Pierce ECL Plus (Thermo Scientific) on ECL hyperfilm.

### Behavioral Assays

All behavioral tests were performed using adult knockout males and their WT controls. To evaluate learning and memory, animals were subjected to the modified Y maze test, the open field test, the contextual/trace fear conditioning and the Morris water maze.

*The Y maze and modified Y maze tests* were performed as previously described (Rzem et al., 2015). Briefly, this test was used to assess spatial working memory. The Y maze was made of three identical opaque arms. Mice were placed into a start arm for 5 min. A total number of arm entries and arm alternation were recorded. In the modified Y maze test, mice underwent two consecutive trials. In the first trial only two arms were accessible. The mouse was placed in the Y maze and allowed to explore the two accessible arms during 10 min. In the second trial, after a 30 min inter-trial interval, the third arm was opened, and the mouse placed back into the Y maze during 5 min with access to all three arms. Mice were video tracked (Ethovision 6.1, Noldus; Wageningen, Netherlands) and the time spent in the novel versus familiar arms, the latency to enter the novel arm and the number of entries into the novel versus familiar arms were assessed.

*The open-field test* was used to assess a non-forced ambulation as mice could move freely without any influence of the examiner. Mice were placed in a square arena (60 × 60 cm) and video tracked (Ethovision) for 20 min. The total distance covered by the animals, the proportion of time spent in the center versus the periphery and the average speed of movement were measured. This test was conducted on two consecutive days.

*The fear conditioning* apparatus (Bioseb, Vitrolles, France) consisted in a square box (25 × 25 × 25 cm) containing an electrifiable grid floor placed on a pressure plate, a sound and a luminous source. Freezing behavior is recorded and analyzed by the software “Freezing” (Bioseb). For both contextual and trace fear conditioning protocols, mice were tested on two consecutive days. For the contextual fear conditioning protocol, on day 1, mice were placed in box with black walls and a steel grid floor (training context). After 2 min of habituation to the context, a tone was presented for 30 s and a mild foot shock was administrated the last two seconds of the tone presentation. After an inter-trial interval of 30 s, mice were exposed to two additional conditioning cycles (3 in total) and left during 30 s in the context chamber before being placed back in their home cage. On day 2, mice were replaced in the same context for 4 min and freezing behavior was recorded. For the trace fear conditioning protocol, on day 1, mice were placed in the box in the training context. After 2 min of habituation to the context, a tone was presented for 30 s and a 2 s mild foot shock was administrated with a trace interval of 15 s. After an inter-trial interval of 30 s, mice were exposed to four additional conditioning cycles (5 in total) and left during 30 s in the context before being placed back in their home cage. On day 2, mice were replaced for 4 min in a new context (white round shape wall, smooth floor). After 2 min of habituation to the context, a tone was presented for 3 min and freezing behavior was recorded.

*The Morris water maze* was used to assess spatial learning and memory (Morris, 1984). Water maze was made of a round pool with a diameter of 113 cm virtually divided into four quadrants (North, South, West and East) and filled with water (26°C). Several visual cues were placed around the pool and a platform was placed at the center of the North-East quadrant of the pool. The time latency to reach the platform, the swim speed, and the time spent in each quadrant were measured (Boucherie et al., 2017).

*The elevated plus maze* test was used to assess anxiety. Mice were placed in an elevated plus maze consisting of two opposing open arm (exposed place) and two opposing closed arm (safer place). Time spent in each arm and distance covered was recorded by a video tracking system (Ethovision) for 5 min (Boucherie et al., 2017).

*The radial maze* test was performed according to the procedure described by Crusio et al. (1993). In brief, we used a maze made of gray plastic with eight identical arms with a central part measuring 15 cm in diameter. Each arm was 27 cm long, 9 cm high and 5 cm wide. A food cup (2.5 cm deep and 2.5 cm in diameter) was inserted at the distal end of each arm. Forty-eight hours prior to training the mice were deprived of food and kept at 80–90% of the pre-test body weight during the 12-day protocol. After two 10 min habituation trials, 24 h apart, with free access to all arms the animals were trained on ten consecutive days, one trial per day. Animals were video-tracked starting day 4 up to day 10. For each animal a specific subset of four arms was randomly selected and designated as the reward set. Only these arms contained a food reward during training. Trials were terminated after the animal had eaten all four rewards or after a maximum of 15 min, whatever came first. A reference memory error was noted if an animal entered an arm that never contained a food reward. A working memory error was noted if an animal entered an arm (rewarded or not) previously visited during the same trial.

*The rotarod test* was performed to assess the ability of mice to keep their balance and coordination on a rotating rod (Bioseb). The time (latency) taken for the mice to fall off the rod rotating under continuous acceleration was measured (Audouard et al., 2013).

“*Physiocages*” (Panlab-Bioseb, Vitrolles, France) were used to measure individual basal activity. Rearing and locomotor activities were measured during 48 h, after 24 h of habituation (Poekes et al., 2017).

### Primary Hippocampal Cell Culture

Coverslips were coated with poly-D-lysine for 3 h in incubator at 37°C and then washed two times with PBS. P0/P1 pups were sacrificed. Brains were removed and placed in sterile and cold HBSS. After meninges removal, hippocampi were carefully removed and put into a new HBSS solution where they were mechanically dissociated by trituration. The tube was left undisturbed for 2 min to allow cell debris to settle down. The supernatant was transferred in a new tube with FBS and centrifuged 500 rpm for 5 min. The supernatant was re-suspended in 1 ml of cell neuronal culture medium (Neurobasal medium supplemented with glutamine, B27, and penicillin-streptomycin). Cells were plated on coverslips and 2 h later, the medium was replaced by fresh one. Cells were maintained at 37°C in a humidified atmosphere of 5% CO_2_ during 15 days.

### Brain Slice Preparation

Animals were sacrificed by cervical dislocation and their brains were quickly removed and placed in ice-cold artificial cerebrospinal fluid (ACSF) composed of (mM): 126 NaCl, 3 KCl, 2.4 CaCl_2_, 1.3 MgCl_2_, 1.24 NaH_2_PO_4_, 26 NaHCO_3_, and 10 glucose (bubbled with 95%O_2_-5% CO_2_%). Cerebellum and frontal cortex were removed. Brains were mounted onto a LeicaVT1200 vibratome, and sagittal sections (or horizontal section for LTD measurements) of a thickness of 350 μm were cut in ice-cold ACSF to obtain dorsal hippocampus (or ventral for LTD). Slices recovered in oxygenated ACSF at 32°C for at least 1 h before use.

### Field Potential Recordings

Mouse brain slices were transferred to the recording chamber and continuously perfused with oxygenated ACSF (2 ml/min) at 30°C. Excitatory postsynaptic potentials (EPSP) were evoked through a bipolar stimulating electrode which was placed in the Schaffer collaterals (SC) and recorded by the AxoClamp 2B (Axon Instruments, United States) amplifier through a glass electrode which was back-filled with 2 M NaCl and placed in the CA1 region (stratum radiatum). Stimuli consisted of 100 μs pulses of constant currents with intensity adjusted to produce 35% of the maximum response every min. After placement of the electrodes, responses were stabilized for 30–60 min (one stimulation per min). Responses were digitized by Digidata 1322A (Axon Instruments, United States) and recorded to a computer using WinLTP software (Anderson and Collingridge, 2007). LTP was induced by applying either a theta burst stimulation (TBS) consisting in four trains of five pulses (100 Hz) separated by a 200 ms interval or a 1 s high frequency stimulation (HFS) at 100 Hz. LTD was induced chemically by applying 50 μM dihydroxyphenylglycine (DHPG) for 20 min. All LTD experiments were done in presence of 50 μM picrotoxin and 5 μM L689560 (trans-2-carboxy-5,7-dichloro-4-phenylaminocarbonylamino-1,2,3,4-tetrahydroquinoline, a very potent antagonist of the N-methyl-D-aspartate receptor (NMDAR) (Tidball et al., 2017). Slopes of field excitatory postsynaptic potentials (fEPSP) responses were expressed normalized to the pre-treatment baseline, defined as 100%.

### Electrophysiology of Cultured Hippocampal Neurons

Patch–clamp recordings of hippocampal neurons were carried out at room temperature, using an EPC-9 amplifier controlled by Patchmaster software (HEKA Elektronik, Lambrecht, Germany). An AgCl wire was used as a reference electrode. Solutions were applied to the cells via a homemade gravity-fed perfusion system, connected by a 5-way manifold, to a RC25 perfusion chamber (Warner Instruments, Hamden, CT, United States). The patch pipettes were pulled with a resistance of 4–7 MΩ using a DMZ-Universal Puller (Zeitz Instruments, Munich, Germany). Series resistances were compensated (75–90%) and periodically monitored. For current–clamp analysis, cells with a resting membrane potential ≥-50 mV were discarded.

For voltage–clamp recordings [except for spontaneous excitatory postsynaptic currents (sEPSC) recordings], the following extracellular solution was used (in mM): 140 NaCl, 5 KCl, 1 CaCl_2_, 1 MgCl_2_, 10 glucose, and 10 HEPES, pH 7.4. The pipette solution had the following composition (in mM): 140 CsCl, 10 EGTA, 0.3 Mg_2_ATP, 0.3 CaCl_2_, and 10 HEPES, pH 7.25. To prevent network activity, the experiments were performed in the presence of 1 μM tetrodotoxin (TTX), 30 μM picrotoxin, 10 μM bicuculline methiodide, 10 μM 6-cyano-7-nitroquinoxaline-2,3-dione disodium (CNQX) and 10 μM D-(-)-2-Amino-5-phosphonopentanoic acid (D-AP5) in the superfusate. Moreover, to prevent the activation of capacitative Ca^2+^ entry upon store depletion, the PLC inhibitor 1-[6-[((17β)-3-Methoxyestra-1,3,5[10]-trien-17-yl)amino]hexyl]-1H-pyrrole-2,5-dione (U-73122, 5 μM) was added in the extracellular medium. sEPSC were recorded at a holding potential of -60 mV. The extracellular solution consisted (in mM) of 160 NaCl, 2.5 KCl, 10 HEPES, 10 glucose, and 2 CaCl_2_, to which 30 μM picrotoxin and 10 μM bicuculline methiodide were added. The pipette solution had the following composition (in mM): 130 K-gluconate, 10 KCl, 1.1 EGTA, 10 HEPES, 1 MgCl_2_, 2 Na_2_ATP, 0.1 CaCl_2_, and 5 QX314Br to block Na^+^ currents (pH 7.2). For current–clamp recordings, the pipette solution had the following composition (in mM): 135 KCl, 1 K_2_ATP, 1 MgATP, 2 EGTA, 1.1 CaCl_2_, 5 glucose, and 10 HEPES (pH 7.2). The extracellular solution contained (in mM): 160 NaCl, 2.5 KCl, 2 CaCl_2_, 2 MgCl_2_, 10 glucose, 10 HEPES (pH 7.4), 1 μM TTX, 30 μM picrotoxin, 10 μM bicuculline methiodide, 10 μM CNQX, 10 μM D-AP5, and 5 μM U-73122 (Patrich et al., 2016). Detection of sEPSC events was performed using Clampfit.

### Calcium Imaging

Calcium measurements were performed as previously described (Gailly et al., 1993; De Backer et al., 2002). Briefly, calcium imaging was performed on DIV13-15 hippocampal cultures. Cells were loaded with 1 μM Fura-2-AM (Molecular Probes) in Krebs solution [contained (mM): 135 NaCl, 5.9 KCl, 2 CaCl_2_, 1.2 MgCl_2_, 10.6 Hepes Na^+^, and 10 glucose (pH 7.3)] for 30 min at room temperature. Fura-2 loaded cells were excited alternatively at 340 and 380 nm and fluorescence emission was monitored at 510 nm using an AxioCam MRm camera (Zeiss) coupled to an inverted microscope (Axiovert 200 M Zeiss, objective 20X Fluar N.A. 0.75). Concentration of intracellular calcium ([Ca^2+^]_i_) was calculated from the ratio of the fluorescence intensities excited at the two wavelengths and after cell calibration, Δ[Ca^2+^] measurements were calculated by subtracting the resting [Ca^2+^]_i_ value from the [Ca^2+^]_i_ peak amplitude. For each experiment, the [Ca^2+^]_i_ values measured on individual cells were averaged for all cells studied on one coverslip.

### RNA Extraction and q-Real Time PCR

RNA expression in hippocampus or cortex was evaluated after total RNA was purified using Trizol method. RT-qPCR primers sequences used were as follows:

*Trpc1*: Fw: CAGAAGGACTGTGTGGGCAT Rv: CAGGTGCCAATGAACGAGTG
*Trpc2*: Fw: ACTTCCTGGACGTGGTCATC Rv: CTGAGCATGCTGGTGACAGT
*Trpc3*: Fw: TGGATTGCACCTTGTAGCAG Rv: ACGTGAACTGGGTGGTCTTC
*Trpc4*: Fw: AAGCCAAGTGGAGAGAAGCA Rv: ATCGGAGCTGGAGACACACT
*Trpc5*: Fw: GCTGAAGGTGGCAATCAAAT Rv: AAGGTTGCTTCTGGGTGAGA
*Trpc6*: Fw: CCTCCCTAATTGAAACCAGCA Rv: GATTGCCAGCATTCCAAAGT
*Trpc7*: Fw: CTGTGAAAACGACCGGAAAT Rv: CCCTCCATTGCTTCATCGTT
*mGlur5*: Fw: CCTTCCCCAAGAGCACAGAG Rv: CATCATAAAGCGCCTTGGGG
*GluA1*: Fw: TCAGAACGCCTCAACGCC Rv: TGTAGTGGTACCCGATGCCA
*GluA2*: Fw: CAGTGCATTTCGGGTAGGGA Rv: TGCGAAACTGTTGGCTACCT
*GluN1*: Fw: CCAGCTTGCTAGGTCACCCT Rv: AGGGTCGTGGGAGGATTGTG
*GluN2A*:Fw: TGCCACAGGGAGCCAGATAAT Rv: TATCCCTGGGAGAACTTGCTTTG
*GluN2B*: Fw: TGTCCACCATTCCTGTTCCC Rv: TCAGGAAAGCCTCGCTCAAA
*Egr-1*: Fw: TGACCAATCCTCCGACCTCT Rv: AGATGGGACTGCTGTCGTTG
*c-fos*: Fw: AGATGTGGACCTGTCCGGTT Rv: TACAGGTGACCACGGGAGTA
*Arc*: Fw: CCTGCTCTTACCAGCGAGTC Rv: CATCCCTTTGGGAGTCAGCC
*Trpc1*α: Fw: GACTACGGTTGTCAGTCCG Rv: ACAGGTGCGACATCCATCG
*Trpc1*β: Fw: GGACTACGGTTGTCAGAAACT Rv: GCTTGGGCAAAGACACATCC
*Trpc1*γ: Fw: GTGCGACAAGGTCCGCAG Rv: CAGGTGCGACATCCATCGTT
*Trpc1*δ: Fw: CGTGCGACAAGGAAACTTATGG Rv: GCTTGGGCAAAGACACATCC
*Trpc1*𝜖: Fw: GACATTCCAGATGTCTGGC Rv: TCCTGAATTCCACCTCCACA

RT-qPCR was performed using 5 μl of cDNA, 12.5 μl of SYBR Green Mix (BioRad) and 250 μM of each primer in a total reaction volume of 25 μl. The reaction was initiated at 95°C for 3 min, and followed by 40 cycles of denaturation at 95°C for 10 s, annealing at 60°C for 1 min and extension at 72°C for 10 s.

Data were recorded on a DNA Engine Opticon RT-qPCR Detection System (BioRad) and cycle threshold (Ct) values for each reaction were determined using analytical software from the same manufacturer.

Each cDNA was amplified in duplicate and Ct values were averaged for each duplicate. To normalize the data, the *gapdh* gene was used as housekeeping gene.

### Drugs

(RS)-DHPG, picrotoxin, 2-Methyl-6-(phenylethynyl)pyridine (MPEP), (S)-(+)-α-amino-4-carboxy-2-methylbenzeneacetic acid (LY367385), L689560, Thapsigargin, D-AP5, U-73122 were obtained from Tocris Bioscience. TTX, bicuculline, QX314Br and CNQX were obtained from Hello Bio and OH-tamoxifen from Sigma. Pico145 was synthesized as previously described and stored at 10 mM in DMSO (Rubaiy et al., 2017b). All drugs were prepared as stock solutions according to the supplier’s recommendations and stored at -20°C until use.

### Statistical Analyses

Data are expressed as mean ± standard error of mean (SEM). For each set of experiments, each n represents one slice (for electrophysiology), one coverslip (for calcium imaging) and one animal (for behaviour testing). Statistical significance was assessed by Student’s *t*-test or two-way analysis of variance (ANOVA) tests for comparisons. Statistical significance was fixed to *p* < 0.05.

## Results

### Generation of TRPC1-Null Mice, and Analysis of TRPC1 Expression in the Mouse Brain

**FIGURE 1:**
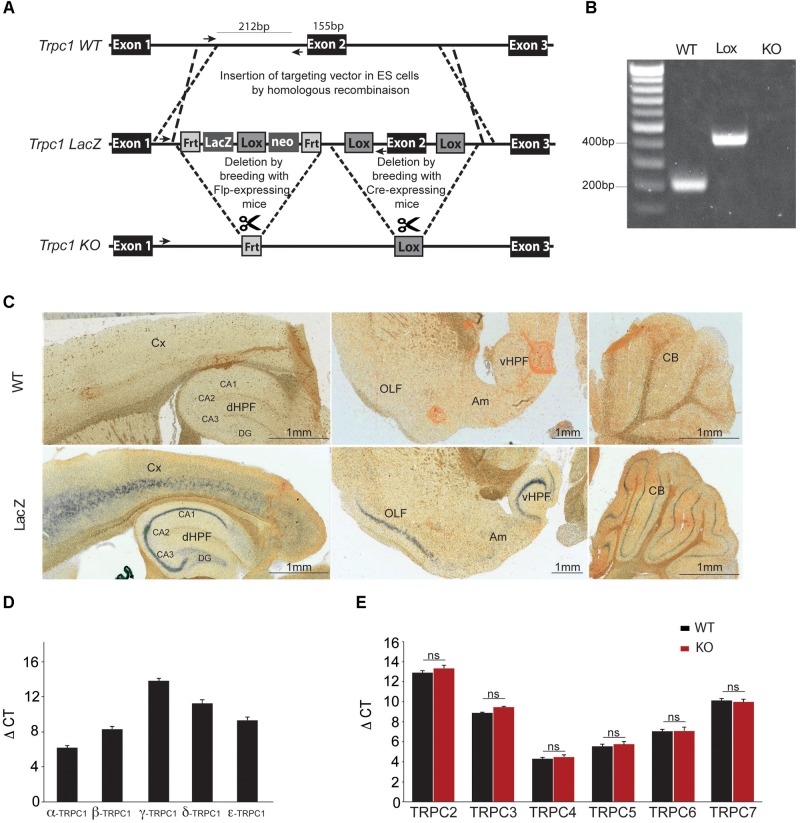
Generation and characterization of the *Trpc1^-/-^* mice. **(A)** Construction scheme of *Trpc1*-LacZ reporter gene and *Trpc1* knockout allele (KO). Sketch 1 shows the wildtype locus (WT). Crossover lines indicate the regions of homologous recombination. Arrows show the sequence targeted by the primers for the PCR. Sketch 2 shows the *Trpc1* gene with the targeting vector, the *LacZ* reporter cassette flanked by *Frt* recombination sites and the exon 2 flanked by *lox* recombination sites. Sketch 3 shows the targeted *Trpc1* locus after removal of *LacZ* by FLP recombination and the disrupted gene after Cre recombination. **(B)** PCR genotyping of *Trpc1^wt/wt^* (212 bp fragment), *Trpc1^lox/lox^* (inserted lox sequence: 420 bp fragment), and *Trpc1^-/-^* (KO allele where the reverse primer site is not present). **(C)** Representative images of β-gal staining of *Trpc1^wt/wt^* and *Trpc1^lacZ/lacZ^* mice sagittal brain sections. CA1-CA3, cornu ammonis regions; Cx, cortex; DG, dentate gyrus; dHPF, dorsal hippocampus formation; vHPF, ventral hippocampus formation; Am, amygdala; OLF, olfactory cortex area; and CB, cerebellum. **(D)** RT-qPCR analysis of *Trpc1* splice variants [*n* = 3, *F*_(5,10)_ = 200.9; *p* < 0.0001; one-way ANOVA; and post-test Newman–Keuls comparing all pairs of columns: *p* < 0.001 except β vs. ε: *p* < 0.01]. **(E)** RT-qPCR analysis of TRPC isoforms in hippocampi from *TRPC1^wt/wt^* (WT) and *Trpc1^-/-^* (KO) mice. ΔCt represents the difference in cycle threshold detection of the mRNA of interest minus the mRNA of the internal control *gapdh* [*n* = 3; *F*_(12,30)_ = 212.8, *p* < 0.001; one-way ANOVA; and post-test Newman Keuls *p* > 0.05].

Genetic ablation of the *Trpc1* gene was achieved by removing the exon 2 genomic region encoding the amino acids 692-846 corresponding to the putative sequence of the cytoplasmic N-terminal domain (**Figure 1A**). The genetic modification induced a frame shift and a stop codon after the deleted fragment at the position 878 in exon 3. The targeting construct was made using homologous recombination. Deletion of exon 2 catalyzed by the Cre-recombinase was confirmed by RT-PCR and sequencing of the amplicon that spanned the deleted region of the gene (**Figure 1B**). To evaluate where TRPC1 is expressed in the brain, we used a genetically modified mouse line, where the *lacZ* reporter gene was expressed under the control of *Trpc1* promoter (**Figure 1A**). *LacZ* expression was detected using a ß-galactosidase enzymatic reaction on brain sagittal sections of adult mice (**Figure 1C**). The staining was found in the cortex, the cerebellum, the amygdala, the olfactory region and in the dorsal and ventral hippocampus. In the latter structure, it was particularly abundant in CA1 to CA3 regions. Among the five splice variants of TRPC1, TRPC1α was the most expressed in the hippocampus (**Figure 1D**). The mRNA expression of TRPC2, 3, 4, 5, 6, and 7 remained unchanged in hippocampi from *Trpc1^-/-^* mice, suggesting that the ablation of TRPC1 was not compensated by overexpression of these related genes (**Figure 1E**).

### Deletion of the Trpc1 Gene Impairs Fear Memory Formation but Not Innate Fear Behavior

**FIGURE 2:**
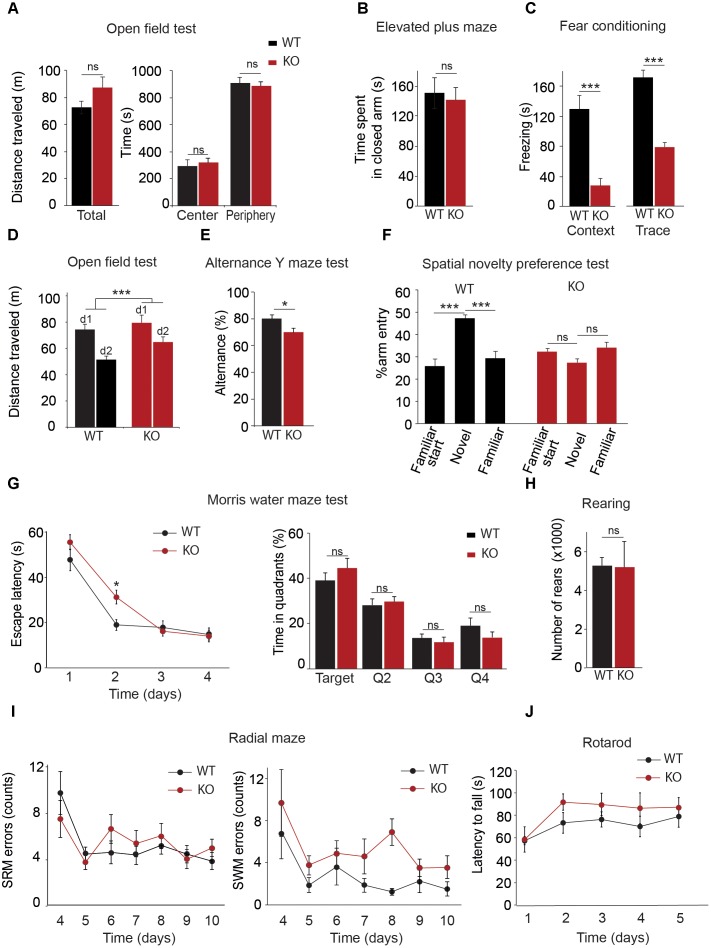
Behavioral characterization of *Trpc1^-/-^* mice (KO). **(A)** Open field test. Left panel: Distances traveled [*n* = 11; WT: mean = 7.34 ± 0.48 m; *Trpc1^-/-^*: mean = 8.7 ± 0.78 m; and *n* = 11, *t*_(20)_ = 1.49, *p* = 0.15, *t*-test]. Right panel: Time spent in central and peripheral areas during a 20 min period [*n* = 11; *F*_(4,40)_ = 0.32; *p* = 0.57; two way ANOVA]. **(B)** Elevated plus maze. Time spent in closed arms during a 5 min period [WT: *n* = 13; mean = 151 ± 21 s; *Trpc1^-/-^*: *n* = 15; mean = 141 ± 17; *t*_(26)_ = 0.36; *p* = 0.72; *t*-test]. **(C)** Fear conditioning tests. Freezing time periods the day after contextual (left) or trace (right) [conditioning contextual fear conditioning: WT: *n* = 17; mean = 131 ± 18 s; *Trpc1^-/-^*: *n* = 10; mean = 28 ± 11 s; *t*_(25)_ = 4.085; *p* = 0.0004; *t*-test; trace fear conditioning: WT: *n* = 5; mean = 163 ± 8 s; *Trpc1^-/-^*: *n* = 8; mean = 75 ± 12 s; *t*_(11)_ = 5.22; *p* = 0.0003; *t*-test]. **(D)** Open field tests performed 2 days in a row. Distances traveled during a 20 min test. [WT: *n* = 14; mean = –29.8 ± 3.2%; *Trpc1^-/-^*: *n* = 17; mean = –17.1 ± 3%; *t*_(2,29)_ = 2.828; *p* = 0.0084; *t*-test]. **(E)** Alternation Y maze test. Proportion of alternation in visiting the different arms (WT: *n* = 7; mean = 80.1 ± 2.7%; *Trpc1^-/-^*: *n* = 12; mean = 70.1 ± 3%; *t*_(17)_ = 2.26; *p* = 0.037; *t*-test). **(F)** Spatial novelty preference test (modified Y maze). Percentage of entries in the novel, in the starting and in the familiar arms [WT: *n* = 7; mean = 45.9 ± 1.8%; *Trpc1^-/-^*: *n* = 5; mean = 28.8 ± 1.9%; *F*_(3,18)_ = 15.21; *p* = 0.001, one way ANOVA; Newman–Keuls *p* < 0.001]. **(G)** Morris Water maze test. The average escape latencies, (i.e., the time required for WT and *Trpc1^-/-^* mice to reach the platform) on the training day (day 1) and on the three consecutive trial days are shown on the left panel [*n* = 13, *F*_(2,72)_ = 2.86; *p* = 0.04; two way ANOVA, Bonferroni: day 2, WT vs. *Trpc1^-/-^ p* < 0.05; other days WT vs. *Trpc1^-/-^ p* > 0.05]. The percentage of time the mice spent in each quadrant (targeted quadrant and Q2, Q3, and Q4) of the platform arena on the fifth day of testing (once the platform is removed) is shown on the right panel mice [*n* = 13, *F*_(8,96)_ = 11.2, *p* < 0.0001, one way ANOVA, Newman–Keuls, WT vs. *Trpc1^-/-^ p* > 0.05]. **(H)** Eight-arms radial maze performed 7 days in a row after 3 days training. Left panel: number of spatial reference memory (SRM) errors [*n* = 8–12, *F*_(1,108)_ = 4.28, *p* > 0.05; two way ANOVA]. Right panel: number of spatial working memory (SWM) errors [*n* = 8–12, *F*_(1,108)_ = 5.7, *p* = 0.03; two way ANOVA]. **(I)** Number of rearings measured by infrared detection of a 48 h period [WT: *n* = 8; mean = 5265 ± 423 rearings/24 h; *Trpc1^-/-^*: *n* = 5; mean = 5188.6 ± 1323 rearings/24 h; *t*_(11)_ = 0.066; *p* = 0.95; *t*-test]. **(J)** Time latencies to fall off the rotating rod [WT: *n* = 8; *Trpc1^-/-^*: *n* = 5; *F*_(2,44)_ = 0.52; *p* = 0.72; two way ANOVA]. ^∗^*p* < 0.05; ^∗∗∗^*p* < 0.001; and ns, not significant.

TRPC1 has been shown to heteromultimerize with TRPC4 and/or TRPC5 (Strubing et al., 2001; Goel et al., 2002; Hofmann et al., 2002). TRPC4 and TRPC5 isoforms are expressed in the hippocampus but also in the amygdala and both isoforms have been involved in innate fear (Riccio et al., 2009, 2014). We therefore investigated the possible role of TRPC1 in the process. We first tested the performance of the mice in an open field brightly illuminated (100 lux). The distance covered was measured for 20 min. WT and *Trpc1^-/-^* mice had a similar activity (**Figure 2A**) and spent a similar proportion of time in the periphery vs. in the center of the arena, suggesting a similar sensitivity to innately aversive stimuli. This was corroborated by the elevated plus maze paradigm. In this test, mice explore a novel environment and usually tend to avoid the exposed/open arms, preferring the closed/secure arms. Both genotypes behaved similarly, suggesting the absence of propensity to anxiety (**Figure 2B**).

The fear conditioning test was used to investigate associative learning abilities. The first day of the test, mice were exposed to a neutral conditional stimulus (the environment of the chamber for the contextual conditioning or an auditory tone for the trace conditioning) and learned to associate it with an aversive unconditional stimulus (a mild electrical foot shock appearing synchronously to the conditional stimulus in the contextual fear test or after 8 s delay in the trace conditioning). Pairing conditional and unconditional stimuli was repeated three times. Twenty-four hours after the training, mice were tested for contextual and trace fear memory consolidation. Freezing time was considered as an indicator of fear memory. In both tests, we found a significant difference between WT and *Trpc1^-/-^* mice (**Figure 2C**).

These results thus contrast with those obtained with *Trpc4^-/-^* mice and *Trpc5^-/-^* mice that presented a decreased innate fear response but a normal conditioned fear memory (Riccio et al., 2009, 2014). This suggests a role of TRPC1 in the hippocampus, which is necessary for learning the tone–shock association (Curzon et al., 2009). We therefore investigated behaviors more specifically involving hippocampal structures.

### Deletion of the Trpc1 Gene Impairs Spatial Working Memory

Spatial memory was first investigated by comparing the spontaneous activity of the mice 2 days in a row. The first day, no differences were found between the two genotypes (see above). As expected, the second day, both WT and *Trpc1^-/-^* mice decreased their activity, suggesting a habituation to their environment (**Figure 2D**). However, the decrease of activity was significantly more important for WT than for *Trpc1^-/-^* mice, suggesting a deficit in spatial memory in the latter. This was further studied using maze tests. Mice usually prefer to explore a new arm of a maze rather than returning to one that was previously visited. This behavior can be quantified by measuring the alternation of visits the different arms in a Y maze. As shown in **Figure 2E**, spontaneous alternation was significantly decreased in *Trpc1^-/-^* compared to WT mice [WT: *n* = 7; mean = 80.1 ± 2.7 %; *Trpc1^-/-^*: *n* = 12; mean = 70.1 ± 3 %; *t*_(17)_ = 2.26; *p* = 0.037; *t*-test]. A modified Y maze (MYM) protocol was used to further evaluate the effect of *Trpc1* on spatial working memory. During the exposure phase, the animals were allowed to explore two arms of the maze for 10 min while the access to the third arm was blocked. As shown in **Figure 2F**, during the re-exposition 30 min later, WT mice exhibited a significant increase of arm entries percentage in the novel arm of the Y maze over the two familiar arms. This preference for the novel arm was not observed for *Trpc1^-/-^* mice, suggesting an impairment in spatial working memory in *Trpc1^-/-^* animals. The Morris water maze (MWM) has been designed to assess spatial reference memory. In this test, mice learn to locate a hidden platform using visual cues and the escape latency to reach the platform is measured. During the first trial (when the platform is visible, at training day 0), both WT and *Trpc1^-/-^* mice reached the platform in about 50 s. The swimming speed was also similar, suggesting the absence of any major locomotion defect. Then mice learn the location of the platform and the latency to reach it decreases from day to day. As shown in **Figure 2G**, the escape latency was significantly higher in *Trpc1^-/-^* mice only the first day of the test but plateaued at the same level after day 2. On day 4, the platform was removed and WT and *Trpc1^-/-^* mice exhibited a similar preference for the quadrant where the platform had been present, suggesting that spatial reference memory was intact in *Trpc1^-/-^*. Finally, we used the 8-arms radial maze to study both spatial reference and working memories in the same test. In this assay, mice progressively learn to discriminate between arms of the radial maze that contain a food reward and arms that do not. Entering into a never-rewarded arm constitutes a reference memory error. Entering into an arm that has already been visited and is no longer rewarded constitutes a working memory error. As shown in **Figure 2I**, *Trpc1^+/+^* and *Trpc1^-/-^* mice learned to locate rewarded arms and we observed no difference in spatial reference memory. However, *Trpc1^-/-^* mice made significantly more spatial working memory errors than *Trpc1^+/+^* mice.

### Deletion of the Trpc1 Gene Does Not Impair Motor Coordination and Learning

Finally, we compared motor abilities of WT and *Trpc1^-/-^* mice. Mean motor activity was measured on sensor platforms and rearing behavior was monitored by infrared detection (**Figure 2H**). No circadian differences could be detected between the two genotypes. Motor coordination and balance were evaluated 5 days in a row by the rotarod test. Performances were similar between genotypes (**Figure 2J**). These findings suggest that motor coordination and learning are not affected by the ablation of TRPC1.

### Trpc1 Is Required for Novelty-Induced Expression of the Immediate Early Gene (IEG) Egr-1 in Hippocampus but Not in Cortex

**FIGURE 3:**
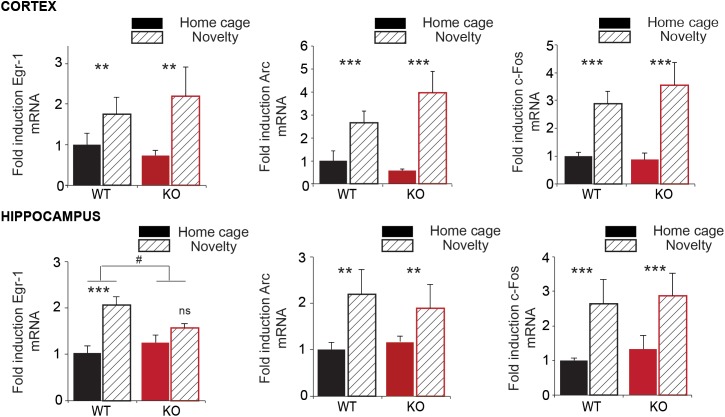
Expression of immediate early genes (IEG) in the cortex and in the hippocampus. RT-qPCR analysis of *Egr-1*, *Arc*, and *c-Fos* expression in the cortex and in the hippocampus of animals exposed (Novelty) or not (Home cage) to a new environment for 45 min. Transcription is measured using *gapdh* as an internal control (ΔCt). The environment-related induction is calculated as 2^-Δ*Ct*^ and expressed in proportion of the expression of the gene of interest in WT in home cage condition. Statistical significance between home cage and novelty: ^∗∗^*p* < 0.01; ^∗∗∗^*p* < 0.001; and ns, not significant. Statistical significance between WT and *Trpc1^-/-^ mice : ^#^p* < 0.05. [cortex: *n* = 5–7; Two way ANOVA: treatment: Egr-1: *F*_(2,19)_ = 7.73; *p* = 0,01; Arc: *F*_(2,19)_ = 19.18; *p* = 0.0003; c-Fos: *F*_(2,19)_ = 43.34; *p* < 0.0001; interaction: Egr-1: *F*_(2,19)_ = 0.22; *p* = 0.65; Arc: *F*_(2,19)_ = 19.18; *p* = 0.46; c-Fos: *F*_(2,19)_ = 0.34; *p* = 0.56; Hippocampus: *n* = 4–8; Two way ANOVA: treatment: Egr-1: *F*_(2,20)_ = 19.07; *p* = 0.0003; Arc: *F*_(2,19)_ = 8.82; *p* = 0.008; c-Fos: *F*_(2,19)_ = 18.32; *p* = 0.0005; interaction: Egr-1: *F*_(2,20)_ = 4.94; *p* = 0.04 (#); Arc: *F*_(2,19)_ = 0.54; *p* = 0.47; c-Fos: *F*_(2,19)_ = 0.25; *p* = 0.62].

At a cellular level, trace of memory can consist in the activation of the transcription of immediate early genes (Minatohara et al., 2015). We performed RT-qPCR on mRNA extracted from WT and *Trpc1^-/-^* mice exposed (novelty) or not (home cage) to an unknown environment (open field). We analyzed the increase of immediate early genes Arc, Egr-1 (also known as Zif268), and c-Fos in the cortex and in the hippocampus 45 min after exposure to these environments (**Figure 3**). No difference of expression was observed at the basal level between WT and *Trpc1^-/-^* mice. In WT animals, exposure to a novel environment (treatment) induced a 1.5–4 times increase in the transcription levels of the three IEG both in the cortex and in the hippocampus. Moreover, the interaction of the treatment with the mice genotype was not different between WT and *Trpc1^-/-^* mice in the cortex. However, the induction of Egr-1 was significantly blunted in the hippocampus of *Trpc1^-/-^* mice, which clearly points out a specific impairment in hippocampal function.

### Deletion of Trpc1 Alters Synaptic Excitability and Plasticity

**FIGURE 4:**
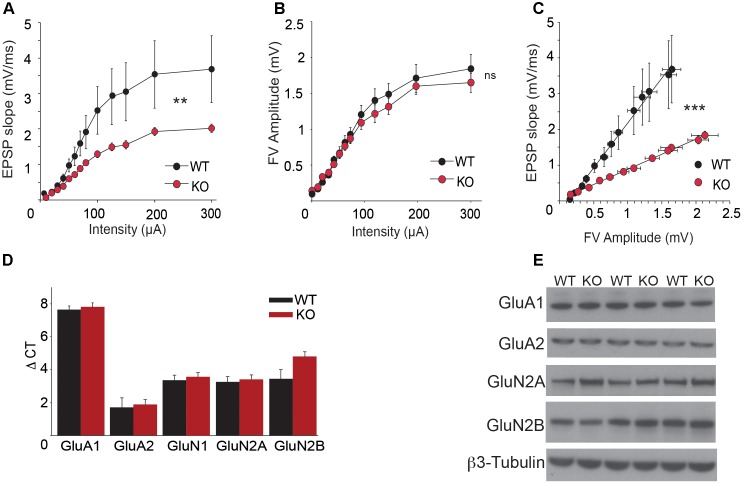
Basal SC-CA1 synaptic transmission in *Trpc1^-/-^* (KO) compared to WT mice. **(A)** The input–output relationship between fEPSP measured in CA1 stratum radiatum and the intensity of SC stimulation [*n* = 10; *F*_(2,216)_ = 2.46; *p* = 0.005; two way ANOVA]. **(B)** Relationship between the amplitude of afferent fiber volley (FV) and the intensity of SC stimulation [*n* = 10; *F*_(2,234)_ = 0.37; *p* = 0.97; two way ANOVA]. **(C)** Relationship between fEPSP measured in CA1 and FV amplitudes [data from **(A)** and **(B)**; *n* = 10; *F*_(2,276)_ = 104.95; *p* < 0.0001; linear regression analysis and ANOVA]. **(D)** RT-qPCR analysis of mRNAs from different AMPAR and NMDAR subunits in hippocampi. ΔCt represents the difference in cycle threshold detection of the mRNA of interest minus the mRNA of the internal control *gapdh* [*n* = 12; *F*_(12,50)_ = 0.37; *p* = 0.83; two way ANOVA, Bonferroni *p* > 0.05]. **(E)** Western-blot analysis of GluA1, GluA2, GluN2A, and GluN2B proteins expression in hippocampi from WT and *Trpc1^-/-^* (KO) mice. ^∗∗^*p* < 0.01; ^∗∗∗^*p* < 0.001; and ns, not significant.

In order to analyze CA3-to-CA1 synapse plasticity, we performed extracellular recordings on hippocampal slices from adult WT and *Trpc1^-/-^* mice. Schaffer collaterals (SC) were stimulated and fEPSP were recorded in the stratum radiatum of CA1 region. We observed a decrease in the relation between the stimulus intensity and the fEPSP slope in slices from *Trpc1^-/-^* mice (**Figure 4A**). However, the amplitudes of afferent fiber volleys (FV) were not reduced (**Figure 4B**), showing a similar axonal spiking of SC. As expected, the relation between the amplitudes of the FV and the fEPSP slopes was thus reduced in *Trpc1^-/-^* mice, pointing to a reduced excitability in the mutant mice (**Figure 4C**). The expression levels of α-amino-3-hydroxy-5-methyl-4-isoxazole propionic acid receptor (AMPAR) and NMDAR subunits were similar in the hippocampus of WT and *Trpc1^-/-^* mice, suggesting that the reduced excitability was not due to a decreased number of receptors in postsynaptic elements (**Figures 4D,E**).

**FIGURE 5:**
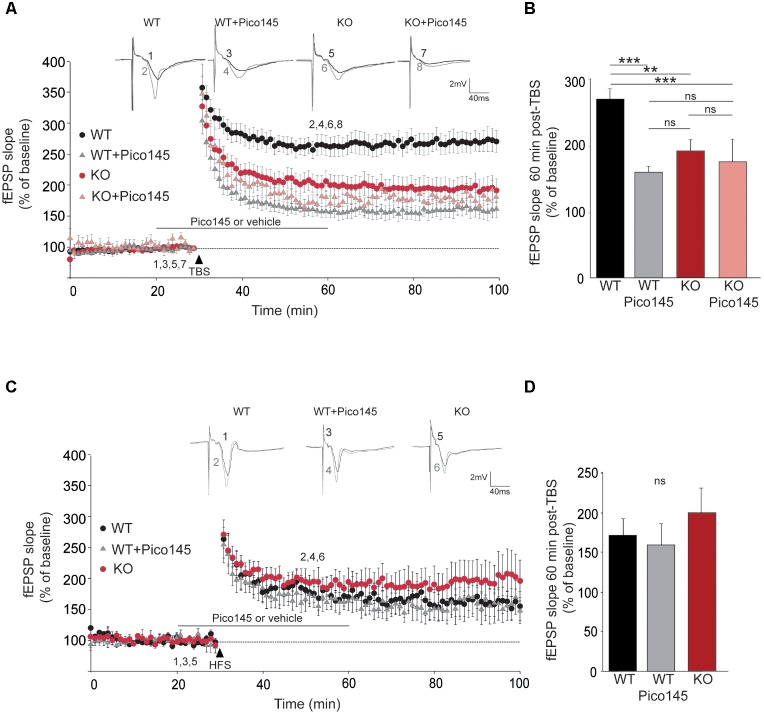
LTP in hippocampal SC-CA1 pathway is impaired in *Trpc1^-/-^* mice. **(A,C)** fEPSP slopes measured before and after theta burst stimulation [TBS, panel **(A)**] or after a high frequency stimulation [HFS, panel **(C)**] in slices from WT and *Trpc1^-/-^* (KO) mice treated or not with 1 nM Pico145 (vehicle DMSO 0.01%) during 40 min. Results expressed in proportion of the baseline response (100%). Upper insets show representative traces of fEPSP before (black) and 30 min after stimulation (gray). **(B,D)** Quantification of the fEPSP slope 60 min after TBS (*n* = 9–12, *F*_(4,38)_ = 11.34; *p* < 0.0001; one way ANOVA Newman–Keuls ^∗∗^*p* < 0.01; ^∗∗∗^*p* < 0.001) or after HFS [*n* = 5, *F*_(3,13)_ = 0.66; *p* = 0,54; one way ANOVA Newman–Keuls]. ^∗∗^*p* < 0.01; ^∗∗∗^*p* < 0.001; and ns, not significant.

To investigate LTP, we stimulated the SC pathway with a TBS consisting of four trains of five pulses (given at 100 Hz) separated by 200 ms. TBS induced a large increase of the fEPSP response size that decayed over the first 20 min to a stable value persisting up to the end of the experiment (Volianskis et al., 2013; Park et al., 2014). The initial decremental potentiation also sometimes referred to as short-term potentiation (STP) reached 350% of the initial response whereas the plateau level of LTP reached 260% on the initial response (**Figure 5A**). We found that STP induction by a TBS was similar in slices from WT and *Trpc1^-/-^* mice but the maintenance of LTP over time was largely reduced in *Trpc1^-/-^* mice (**Figures 5A,B**). Typically, 60 min after the TBS, LTP was reduced by half. We then investigated whether a similar effect could be obtained after acute inhibition of TRPC1 channel by the newly characterized specific inhibitor of TRPC1/4/5 channels, Pico145 (Rubaiy et al., 2017b). Pico145 (1 nM) was perfused 10 min before and 30 min after TBS induction. We observed a normal induction of STP, which did not persist over time (LTP) (**Figures 5A,B**).

The important reduction of LTP contrasts with previous observations showing that LTP is normal in *Trpc1^-/-^*, *Trpc1/4^-/-^*, and *Trpc1/4/5^-/-^* mice (Phelan et al., 2013; Broker-Lai et al., 2017; Li et al., 2018). However, in all these studies, LTP was induced by strong tetanization (HFS consisting in a 1 s train of 100 pulses at 100 Hz). We therefore repeated these measurements and confirmed that LTP induced by HFS was neither altered in *Trpc1^-/-^* vs. WT mice nor affected by 1 nM Pico145 (**Figures 5C,D**).

### mGluR5 Stimulation Induces an Entry of Ca^2+^ Through TRPC1 Channel in Hippocampal Neurons

**FIGURE 6:**
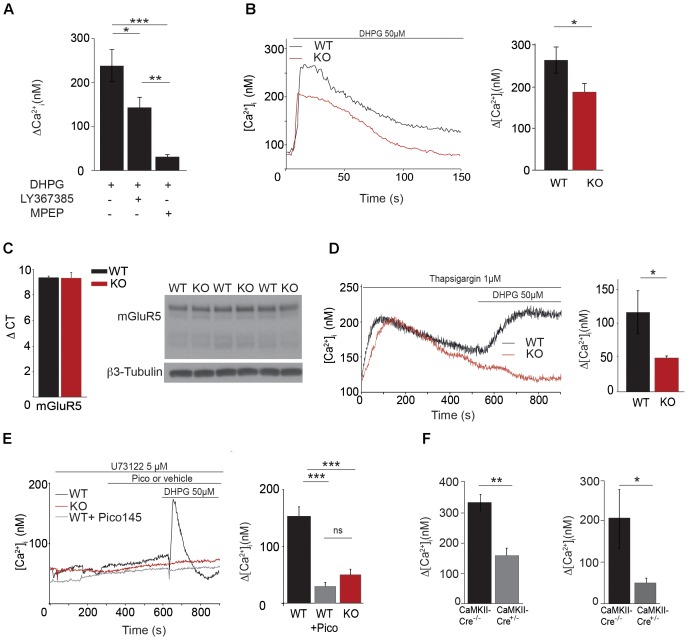
Characterization of [Ca^2+^]_i_ transients through TRPC1 in cultured hippocampal neurons. **(A)** [Ca^2+^]_i_ response to 50 μM DHPG in the presence or in the absence of 10 μM LY367385 or 50 μM MPEP. Δ[Ca^2+^] measurements were calculated by subtracting the resting [Ca^2+^]_i_ value from the [Ca^2+^]_i_ peak amplitude [DHPG: *n* = 5; DHPG + LY: *n* = 6; DHPG + MPEP: *n* = 5; *F*_(3,13)_ = 16.97; *p* = 0.0002; one way ANOVA; Newman–Keuls DHPG vs. DHPG + MPEP: *p* < 0.001; DHPG vs. DHPG + LY: *p* < 0.05; DHPG + LY vs. DHPG + MPEP: *p* < 0.01]. **(B)** Representative traces (left panel) and quantification (right panel) of DHPG-induced responses measured in neurons isolated from WT and *Trpc1^-/-^* mice [WT: *n* = 18 mean = 274 ± 31 nM; *Trpc1^-/-^*: *n* = 21 mean = 18.6 ± 23 nM; *t*_(37)_ = 2.33; *p* = 0.025; *t*-test]. **(C)** RT-qPCR analysis (left panel) and Western blot analysis (right panel) of mGluR5 expression in hippocampi from WT and *Trpc1^-/-^* (KO) mice. ΔCt represents the difference in cycle threshold detection of the mRNA of interest minus the mRNA of the internal control *gapdh* [WT: *n* = 6 mean ΔCt = 9.33 ± 0.13; *Trpc1^-/-^*: *n* = 6 mean ΔCt = 9.28 ± 0.45; *t*(10) = 0,82; *p* > 0.05; *t*-test]. **(D)** Representative traces (left panel) and quantification (right panel) of DHPG-induced responses after treatment with 1 μM thapsigargin in *Trpc1^-/-^* or WT neurons [WT: *n* = 4 mean = 113 ± 30 nM; *Trpc1^-/-^*: *n* = 6 mean = 48 ± 2 nM; *t*_(8)_ = 2.62; *p* = 0.03; *t*-test]. **(E)** Representative traces (left panel) and quantification (right panel) of DHPG-induced responses in the presence of 5 μM U-73122 in *Trpc1^-/-^* neurons (red trace) or in WT treated (pink trace) or not (black trace) with 1 nM Pico145 [WT: *n* = 9 mean = 134 ± 12 nM; *Trpc1^-/-^*: *n* = 8 mean = 49 ± 9 nM; WT + Pico: mean = 32 ± 7 nM; *F*_(3,31)_ = 29.01 *p* < 0.0001; one way ANOVA; Newman–Keuls: WT vs. *Trpc1^-/-^ p* < 0.001, WT vs. WT + Pico *p* < 0.001, *Trpc1^-/-^* vs. WT + Pico *p* > 0.05]. **(F)** Quantification of DHPG-induced Ca^2+^ responses measured in neurons isolated from *Trpc1^lox/-^CaMKII-CreERT2^+/-^* and from *Trpc1^lox/-^ CaMKII-CreERT2^-/-^* mice, pre-treated for 48 h with 1 μM 0H-tamoxifen. Left panel: experiments done in the absence of U-73122 [*Trpc1^lox/-^ CaMKII-CreERT2^-/-^*: *n* = 7 mean = 335 ± 28 nM; *Trpc1^lox/-^ CaMKII-CreERT2^+/-^ n* = 8; mean = 159 ± 28 nM; *t*(13) = 0,001; ^∗∗^*p* < 0.01; *t*-test]. Right panel: experiments done on the presence of U-73122 [*Trpc1^lox/-^ CaMKII-CreERT2^-/-^*: *n* = 5 mean = 206 ± 61 nM; *Trpc1^lox/-^ CaMKII-CreERT2^+/-^*: *n* = 5 mean = 50 ± 8 nM; *t*(8) = 0,035; ^∗^*p* < 0.05; *t*-test]. ^∗^*p* < 0.05; ^∗∗^*p* < 0.01; and ^∗∗∗^*p* < 0.001.

As excitability and LTP were decreased in CA1 glutamatergic synapses from *Trpc1^-/-^* mice, we tested whether TRPC1 channel might be activated by mGluRs in hippocampal neurons. We observed that stimulation of neurons with the selective agonist of group I mGluR DHPG (50 μM) induced an intracellular Ca^2+^ transient that was slightly reduced by pre-treatment with 10 μM LY367385, a specific inhibitor of mGluR1 but completely abolished by 50 μM MPEP, a specific inhibitor of mGluR5 (**Figure 6A**). The response to DHPG was also significantly reduced in neurons from *Trpc1^-/-^* mice (**Figure 6B**) in spite of the fact that the expression of mGluR5 was unchanged (**Figure 6C**). After having emptied the stores of Ca^2+^ with the SERCA inhibitor thapsigargin (1 μM), DHPG still induced a Ca^2+^ response (**Figure 6D**) that was not observed in the absence of Ca^2+^ in the external medium (data not shown). This observation suggested that DHPG induced an entry of Ca^2+^ that was at least partially independent of ER store depletion. Accordingly, after inhibition of the mGluR-induced phospholipase C pathway by 5 μM U-73122, DHPG was still able to induce an entry of Ca^2+^ in neurons from WT animals (**Figure 6E**). This store-independent entry of Ca^2+^ induced by DHPG was not observed in neurons from *Trpc1^-/-^* mice or after inhibition of TRPC1 by 1 nM Pico 145 (**Figure 6E**). Finally, to affect specifically and acutely TRPC1 expression, we made use of a mouse model conditionally expressing TRPC1. We compared neurons isolated from *Trpc1^lox/-^ CaMKII-CreERT2^+/-^* and from *Trpc1^lox/-^ CaMKII-CreERT2^-/-^* mice. These neurons were treated for 48 h with 1 μM OH-tamoxifen to inhibit the expression of TRPC1 in *Trpc1^lox/-^ CaMKII-CreERT2^+/-^* neurons. We observed that the response to DHPG was significantly reduced in these cells (**Figure 6F**). These observations suggest that the acute and specific neuronal inhibition of TRPC1 is sufficient to decrease the mGluR5-induced Ca^2+^ entry.

### mGluR5 Stimulation Modulates Synaptic Excitability Through TRPC1 Channel Activation

**FIGURE 7:**
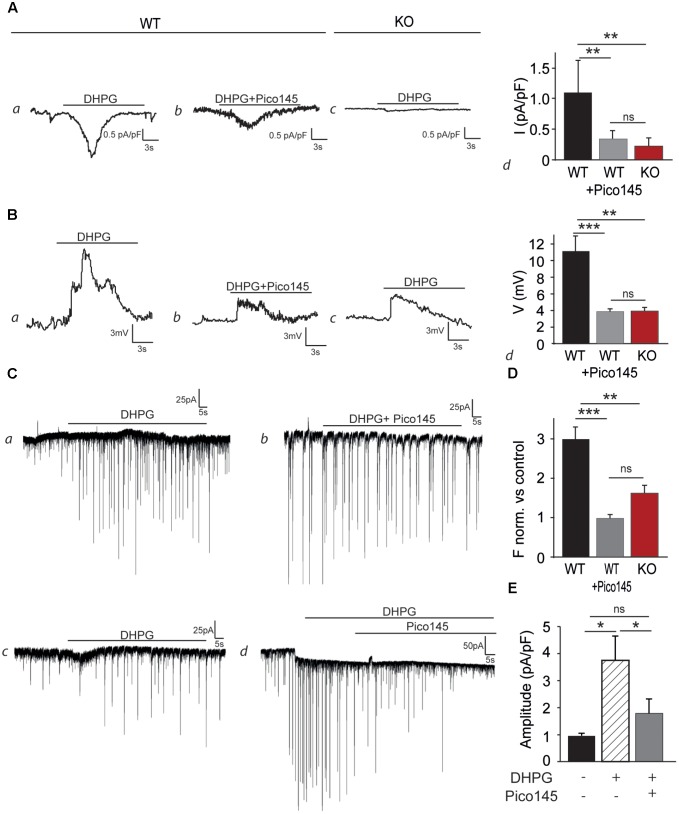
DHPG evokes an inward current and increases the excitability in cultured hippocampal neurons. **(A)** Representative inward current traces recorded at –60 mV, through WT (a,b) and Trpc1*^-^*^/^*^-^* (c) hippocampal neurons, in the presence of 50 μM DHPG and Pico145. Pooled data of whole-cell current evoked by DHPG and Pico145, through *Trpc1^+/+^* and *Trpc1^-/-^* neurons (d) [*n* = 5; *F*_(3,12)_ = 13.79; *p* = 0.0008; one-way ANOVA; Newman–Keuls WT vs. *Trpc1^-/-^*: *p* < 0.01; WT vs. WT + Pico: *p* < 0.001; WT + Pico vs. *Trpc1^-/-^*: *p* > 0.05]. **(B)** Representative current–clamp traces recorded without current injection, through WT (a,b) and *Trpc1^-/-^* (c) hippocampal neurons, in the presence of 50 μM DHPG and 1 nM Pico145, as indicated by the black bar. The resting membrane potentials of recorded neurons have the following values: –66 mV (*a*), –63 mV (*b*), and –64 mV (*c*). Pooled data of voltage traces evoked by DHPG and Pico145, through *Trpc1^+/+^* and *Trpc1^-/-^* neurons (d) [*n* = 5; *F*_(3,12)_ = 10.67; *p* = 0.002; one-way ANOVA; Newman–Keuls WT vs. *Trpc1^-/-^*: *p* < 0.01; WT vs. WT + Pico: *p* < 0.01; WT + Pico vs. *Trpc1^-/-^*: *p* > 0.05]. **(C)** Representative voltage–clamp recordings of sEPSC of cultured hippocampal neurons from WT (*a,b,d*) and *Trpc1^-/-^* (*c*) mice, in the presence of 50 μM DHPG and 1 nM Pico145, as indicated by the black bars. **(D)** Pooled data of normalized sEPSC frequency (vs. controls, experiments *a, b, c*) from WT and *Trpc1^-/-^* neurons (*e*) [*n* = 5; *F*_(3,12)_ = 19.84; *p* = 0.0002; one-way ANOVA; Newman–Keuls WT vs. *Trpc1^-/-^*: *p* < 0.01; WT vs. WT + Pico: *p* < 0.001; WT + Pico vs. *Trpc1^-/-^*: *p* > 0.05]. **(E)** Quantification of the EPSC amplitude (experiment *d*) [*n* = 5; *F*_(3,12)_ = 5.52; *p* = ; one-way ANOVA; Newman Keuls DHPG vs. control: *p* < 0.05; DHPG vs. DHPG + Pico: *p* < 0.05]. ^∗^*p* < 0.05; ^∗∗^*p* < 0.01; ^∗∗∗^*p* < 0.001; and ns, not significant.

As excitability and LTP were decreased in CA1 glutamatergic synapses from *Trpc1^-/-^* mice, we tested whether TRPC1 was involved in mGluR-mediated responses. Hippocampal neurons isolated from newborn (P0/P1) mice and cultured 13–18 days were voltage-clamped at -60 mV. To prevent neuronal activity, experiments were performed in the presence of 1 μM TTX, 10 μM CNQX, 10 μM D-AP5, 30 μM picrotoxin, and 10 μM bicuculline to inhibit Na^+^ voltage-dependent channels, AMPA, NMDA, and GABA receptors, respectively. Moreover, to prevent the activation of store-dependent entry of Ca^2+^, the PLC inhibitor U-73122 (5 μM) was also added in the extracellular medium. In these conditions, stimulation of the wild type cells with 50 μM DHPG induced an inward current of about 45 pA (1.13 ± 0.22 pA/pF, *n* = 5, **Figure 7A**). This response was largely reduced when the cells were perfused with 1 nM Pico145 (0.34 ± 0.06 pA/pF, *n* = 5) and was also significantly smaller in neurons isolated from *Trpc1^-/-^* mice (0.23 ± 0.06 pA/pF, *n* = 5). In current–clamp mode, the mean resting potential of WT neurons, corrected for liquid junction potential (∼5 mV), was –67 ± 4 mV (*n* = 18). Stimulation of the cells with 50 μM DHPG depolarized the cells by about 11 mV (**Figure 7B**). As expected, after treatment of the cells with 1 nM Pico145 or in *Trpc1^-/-^* neurons, the depolarization was significantly decreased although not abolished. To examine the effect of these observations on synaptic excitability, we measured sEPSC of neurons kept in culture and treated with 30 μM picrotoxin, 10 μM bicuculline, and 5 μM U-73122. We observed that stimulation of the cells with 50 μM DHPG increased by a factor of three the frequency of sEPSC. This effect was significantly diminished in neurons treated with 1 nM Pico 145 and in neurons isolated from *Trpc1^-/-^* mice (**Figures 7C**, D). Treatment with 1 nM Pico145 also diminished the amplitude of DHPG-induced EPSC (**Figure 7E**).

### mGluR5 Antagonist (MPEP) Impairs LTP Only in WT Animals

**FIGURE 8:**
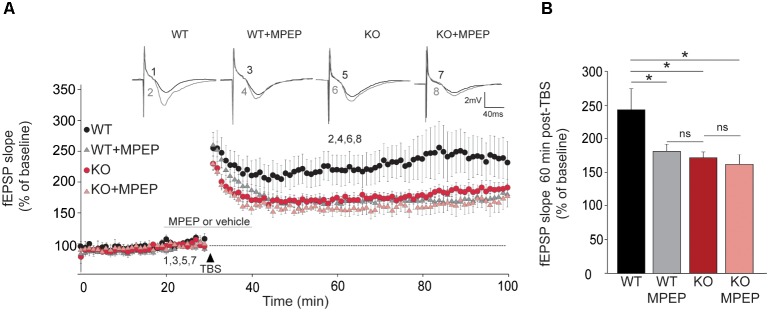
TRPC1-dependent LTP involves mGluR5 in hippocampal SC-CA1 pathway. **(A)** fEPSP slopes measured before and after theta burst stimulation (TBS) in slices from WT and *Trpc1^-/-^* (KO) mice treated or not with 50 μM MPEP during 20 min. Results expressed in proportion of the baseline response (100%). Upper insets show representative trace of fEPSP before (black) and 30 min after TBS (grey). **(B)** Quantification of the fEPSP slope 60 min after TBS [*n* = 7–10 *F*_(4,28)_ = 3.8; *p* = 0.02; one way ANOVA Newman–Keuls ^∗^*p* < 0.05].

To understand the participation in the plateau phase of LTP and to see whether this involves its activation by mGluRs, we compared the effect of 50 μM MPEP on LTP in brain slices from WT and *Trpc1^-/-^* mice. In WT brain slices, 50 μM MPEP significantly reduced the plateau phase of LTP by about half, 1 h after induction. STP was not affected by MPEP. In *Trpc1^-/-^* brain slices, the plateau phase was already reduced (see **Figure 5**) and MPEP did not exert any further inhibition (**Figure 8**).

### DHPG-induced LTD Is impaired in *Trpc1^-/-^* mice

**FIGURE 9:**
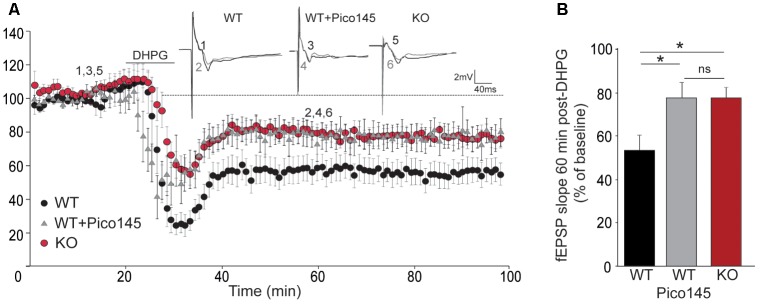
DHPG-induced LTD in hippocampal SC-CA1 pathway is impaired in *Trpc1^-/-^* mice. **(A)** Time-course of fEPSP slopes (mean ± SEM) measured before and after stimulation with 100 μM DHPG in slices from *Trpc1^-/-^* mice (KO) and WT mice perfused or not with 1 nM Pico145 (vehicle DMSO 0.01%) during 40 min. Upper insets show representative traces of fEPSP before (black) and 30 min after DHPG (gray). **(B)** Quantification of the averaged fEPSP slope 60 min after DHPG induction. [*n* = 9 *F*_(3,24)_ = 4.46; *p* = 0.02; one way ANOVA Newman–Keuls ^∗^*p* < 0.05].

LTD consists in a sustained decrease of EPSP and is mediated, at least partially, by endocytosis and decreased surface expression of postsynaptic AMPARs. It relies on rapid dendritic protein synthesis (Huber et al., 2001) but may also be expressed presynaptically (Bolshakov and Siegelbaum, 1994). Agonists of group I mGluRs have been shown to induce a chemical LTD of synaptic transmission in the CA1 region (Palmer et al., 1997; Fitzjohn et al., 1999). Having shown that mGluR5 activates TRPC1, we investigated the possible involvement of TRPC1 in LTD. mGluR-dependent LTD was chemically evoked with 50 μM DHPG. Slices from the ventral part of the hippocampus were used as this part of the hippocampus has a greater ability to exhibit DHPG-induced LTD (Tidball et al., 2017). We observed a reduction of synaptic strength in WT animals that stabilized at around 55% of the pre-stimulation level. The depression was reduced by half in *Trpc1^-/-^* animals and by pretreatment of WT brain slices with 1 nM Pico145 (**Figure 9**).

## Discussion

Several *in vitro* studies showed that TRPC1 can form channel complexes with TRPC4 and TRPC5 but not with TRPC3 and TRPC6, and that all other TRPCs exclusively assemble into homo- or hetero-tetramers within their own subfamilies (Hofmann et al., 2002; Alfonso et al., 2008). Co-immunoprecipitation experiments performed on synaptosomal preparations confirmed the presence of TRPC1/4/5 and TRPC3/6/7 complexes in rat brain (Strubing et al., 2001; Goel et al., 2002). TRPC1 was however shown to also heteromultimerize with TRPC3/6 *in vitro* (Storch et al., 2012) but *in vivo*, this association required the presence of both TRPC1 and TRPC4/5 and seemed to occur only in embryonic brain (Strubing et al., 2003). In adult brain, TRPC1, TRPC4, and TRPC5 are expressed in the amygdala and the hippocampus, two formations critically involved in memory processing (Izquierdo and Medina, 1993; Miller et al., 1998; Riccio et al., 2002). Within the hippocampus, they are present in CA1-3 regions and in the dentate gyrus (Chung et al., 2006), TRPC1 being expressed on both cell somata and dendrites whereas TRPC5 is exclusively located on cell bodies (von Bohlen Und Halbach et al., 2005).

Here, we used a mouse model expressing a β-galactosidase reporter under the control of *Trpc1* promoter and confirmed that TRPC1 was highly expressed in the hippocampus, in particular in the CA1-3 region but also in the dentate gyrus, and at lower levels in the cortex, the olfactory area, the amygdala, and the cerebellum. We generated a genetically modified mouse model in which the *Trpc1* gene was ablated and investigated the behavior of these mice.

*Trpc1^-/-^* mice exhibited a normal innate fear response but an impaired ability in associative learning between conditioned stimulus (CS) and unconditioned stimulus (US) (contextual and trace fear conditioning tests), a process also involving the hippocampus (Curzon et al., 2009). Other tests like the modified Y maze and the radial maze tests pointed out a specific deficit in spatial working memory. The Morris water maze and the radial maze, however, revealed that reference spatial memory was normal in these mice. We also did not observe any impairment in locomotor learning ability. The *Trpc1^-/-^* phenotype thus led us to focus on hippocampal formation and to investigate the cellular mechanisms possibly involved in neuronal excitability and synaptic plasticity (Tonegawa et al., 1996; Tsien et al., 1996; Neves et al., 2008; Bannerman et al., 2014).

We found that TRPC1 is necessary for the response to DHPG, an agonist of group I mGluR. This group of receptors includes mGluR1 and mGluR5, which are coupled to G_q_ /G_11_ G-proteins and activate phospholipase Cβ, resulting in the hydrolysis of PIP_2_ into IP_3_ and DAG (Niswender and Conn, 2010). In hippocampal neurons, the response to DHPG was abolished by MPEP, suggesting a predominant role for mGluR5. The entry of cations triggered by mGluR activation was at least partially independent of the PLC activity and the subsequent release of Ca^2+^ from the stores. DHPG induced a depolarization and increased synaptic excitability. These effects were not observed in *Trpc1^-/-^* neurons. Accordingly, in *Trpc1^-/-^* brain slices, we noted a decreased excitability in SC-CA1 synapses. These results corroborate observations reported recently, showing a reduced synaptic transmission and firing output in CA1 neurons of *Trpc1/4/5^-/-^* mice (Broker-Lai et al., 2017). Of note, the expression levels of AMPA and NMDA receptors were normal in hippocampi from *Trpc1^-/-^* mice and the fiber volleys in the SC were unchanged, suggesting that the number of stimulated fibers and the number of receptors were similar to WT.

Having described the essential role of TRPC1 in mGluR5-mediated responses, we studied its role in plasticity in SC-CA1 synapses. LTP is predominantly triggered by synaptic activation of NMDAR (Park et al., 2014). It indeed requires a rise of intracellular Ca^2+^ arising from influx through NMDAR, or alternatively, L-type Ca^2+^ channels or TRP channels or even from a release of Ca^2+^ from intracellular stores. Many cellular mechanisms modulate LTP, such as stimulation of mGluR expressed presynaptically or postsynaptically (Anwyl, 2009). A chemical LTP can be induced by co-activation of NMDAR and mGluR in hippocampal CA1 neurons (Fujii et al., 2004). The effect is IP_3_-independent. In addition, mGluR activation facilitates activity-dependent LTP in CA1 neurons (Behnisch and Reymann, 1993) and mGluR antagonists impair LTP (Balschun et al., 1999). Among group I mGluRs, mGluR5 has been found in extrasynaptic and perisynaptic sites of CA1 pyramidal neurons whereas mGluR1 is not highly expressed in pyramidal neurons but more on interneurons (Lujan et al., 1996; Ferraguti et al., 1998). mGluR5 is necessary for late-phase LTP in CA1 region (Francesconi et al., 2004; Wang et al., 2016), and mice lacking mGluR5 show a reduced LTP in CA1 region although they present normal LTP in CA3 (Lu et al., 1997). In our *Trpc1^-/-^* mouse model, we observed that TBS-induced LTP was reduced. The sustained phase of LTP is triggered by GluN2A and GluN2B subunits of the NMDAR (Volianskis et al., 2013; Park et al., 2014). Expression of these subunits was normal in the hippocampus of *Trpc1^-/-^* mice and could therefore not explain the reduced sustained phase of LTP. As mentioned above, LTP induced by a strong tetanization (HFS) was reported to be normal in *Trpc1^-/-^*, *Trpc1/4^-/-^*, and *Trpc1/4/5^-/-^* mice (Phelan et al., 2013; Broker-Lai et al., 2017) and we confirm these results in the present study. TBS and HFS may engage different signaling pathways, such as calpain, ERK, or PKA (Zhu et al., 2015), however, TBS tends to better mimic the endogenous theta waves oscillations, the rhythm of which is believed to be critical for temporal coding of synaptic plasticity (Buzsaki, 2002). Interestingly, it has been reported that activation of mGluR5 facilitates LTP generated by a weak tetanization paradigm such as TBS but fails to affect a robust LTP generated by strong tetanization (Balschun et al., 1999). We thus propose that TRPC1 exerts its effect in TBS-induced LTP following its activation by mGluR5. Accordingly, the residual LTP observed in brain slices from *Trpc1^-/-^* mice could not be further reduced by mGluR5 antagonists. It is also interesting to note that the behavioral phenotype of *Trpc1^-/-^* mice is quite similar to the one described for mice lacking mGluR5, which exhibited a decreased learning acquisition in the Morris water maze and a decreased contextual fear-conditioning (Lu et al., 1997). However, it is clear that inhibiting TRPC1 genetically (*Trpc1^-/-^* mice) or pharmacologically (Pico145) affects mGluR5-induced Ca^2+^ entry but not mGluR5-induced Ca^2+^ release from the ER, suggesting that TRPC1 inhibition might only partially affect mGluR5 function.

We show that the DHPG-induced LTD is also decreased in *Trpc1^-/-^* mice. It is not yet clear whether the synaptic depression induced by DHPG in hippocampal CA1 cells arises from pre- and/or post-synaptic mechanisms. In the postsynaptic element, DHPG stimulation is followed by a [Ca^2+^]_i_ increase which, when not associated to a specific electrical stimulation pattern, elicits LTD rather than LTP, because of its smaller amplitude and slower rate; so it can selectively activate the high affinity Ca^2+^-binding proteins [such as calcineurin (PP2B) and PP1] (Lisman, 1989; Collingridge et al., 2010) that mediate rapid postsynaptic protein synthesis which participate in the regulation of AMPAR endocytosis and/or trafficking (Huber et al., 2001; Luscher and Huber, 2010). Several studies show that this rise in [Ca^2+^]_i_ associated to DHPG-LTD is independent from NMDAR, leading us to hypothesize a role of TRPC1 in this Ca^2+^ response. DHPG also depresses excitatory synaptic transmission through changes in presynaptic Ca^2+^ signaling, which indicates either the presence of a retrograde messenger (Watabe et al., 2002) or a presynaptic site of mGluRs and TRPC1 expression. The TRPC1 involvement in pre and/or postsynaptic LTD needs to be further investigated.

TRPC1 has been involved in proliferation of adult hippocampal neural progenitor cells and seems essential in enrichment-induced cognitive enhancement (Li et al., 2012; Du et al., 2017). Histologic examination of brain slices did not reveal any major difference between WT and *Trpc1^-/-^* mice. However, TRPC1 has been shown to modulate the expression of several proteins that could play a role in memory, such as α-internexin and glia maturation factor β (Xing et al., 2016). Moreover, as TRPC1 is known to heteromultimerize with TRPC4 and/or TRPC5, we cannot exclude that, in the absence of TRPC1, these complexes might be differently localized in the cell, even if there expression is unchanged (**Figure 1E**). Therefore, to exclude that our observations could be related to developmental impairment due to the lack of TRPC1, we investigated the effects of 1 nM Pico145, a specific inhibitor TRPC1/4/5 channels. The drug (initially called HC-608) was shown to be highly potent, with subnanomolar IC50 values, and at least 400-fold selective over a wide range of molecular targets including ion channels, receptors, and kinases (Just et al., 2018). We observed that acute inhibition of TRPC1/4/5 perfectly mimicked the lack of TRPC1 channel: it almost abolished mGluR5-induced entry of cations in isolated neurons, inhibited membrane depolarization and excitability triggered by DHPG and reduced TBS-induced LTP and DHPG-induced LTD in brain slices.

Finally, induction of LTP is associated with a rapid transcription of different IEG such as Arc, Egr-1 and c-Fos, which is essential for the transition from short- to long-term synaptic plasticity (Jones et al., 2001). In this process, postsynaptic Ca^2+^ elevation, even modest and transient, seems to play a pivotal role (Murphy et al., 1991; Werlen et al., 1993). Among the IEGs, Egr-1 was shown to increase its activity in CA1 region after contextual learning and fear conditioning (Hall et al., 2000; Lonergan et al., 2010; Veyrac et al., 2014). *In vitro*, TBS-induced LTP of SC-CA1 synapses were also associated with an increased expression of Egr-1 (Roberts et al., 1996). Interestingly, in our *Trpc1^-/-^* mouse model, the association between contextual learning and Egr-1 was significantly blunted in the hippocampus but not in the cortex.

In conclusion, the present study provides evidence that TRPC1 expressed in the hippocampus is essential for the inward current response following activation of mGluR5, which represents a key mechanism in the control of synaptic plasticity. We therefore propose a model in which the glutamate released at SC-CA1 synapses activates mGluR5 receptors localized perisynaptically, which leads to activation of TRPC1-containing channels, allowing an influx of Ca^2+^ and Na^+^ into the postsynaptic element. This would transiently depolarize the cell and modify its excitability, and induce a modest Ca^2+^ transient that could trigger Ca^2+^-dependent processes such as Egr-1 expression. The lack of TRPC1 channel impairs these processes and results in a functional impairment of spatial working memory.

## Author Contributions

SL, RG, FT, and PG designed the study, performed the experiments, interpreted the data, and wrote the paper. ST, OS, FS, AK, XY, AS, and MdC performed the experiments and interpreted the data. NT, TV, RB, and DB critically revised the manuscript. All authors provided final approval for the version of the manuscript submitted for publication and agree to be accountable for the work.

## Conflict of Interest Statement

The authors declare that the research was conducted in the absence of any commercial or financial relationships that could be construed as a potential conflict of interest.
